# Mammalian enabled protein enhances tamoxifen sensitivity of the hormone receptor-positive breast cancer patients by suppressing the AKT signaling pathway

**DOI:** 10.1186/s13062-024-00464-3

**Published:** 2024-03-08

**Authors:** Lifang He, Chuanghong She, Sen Jiang, Zhaochang Qi, Zihao Deng, Likeng Ji, Yukun Cui, Jundong Wu

**Affiliations:** 1 Breast Center, Cancer Hospital of Shantou University Medical College, Shantou, Guangdong China; 2 Laboratory for Breast Cancer Diagnosis and Treatment of Shantou University Medical College, Cancer Hospital of Shantou University Medical College, Shantou, Guangdong China; 3 The Breast Center, People’s Hospital of Jieyang, Jieyang, Guangdong China; 4 Department of Radiology, Cancer Hospital of Shantou University Medical College, Shantou, Guangdong China

**Keywords:** MENA, HR + breast cancer, Drug resistance, Tamoxifen, Organoid

## Abstract

**Background:**

Mammalian enabled (MENA) protein is a member of the enabled/vasodilator stimulated phosphoprotein (Ena/VASP) protein family, which regulates cytoplasmic actin network assembly. It plays a significant role in breast cancer invasion, migration, and resistance against targeted therapy and chemotherapy. However, its role in the efficacy of endocrine therapy for the hormone receptor-positive (HR^+^) breast cancer patients is not known. This study investigated the role of MENA in the resistance against tamoxifen therapy in patients with HR^+^ breast cancer and the underlying mechanisms.

**Methods:**

MENA expression levels in the clinical HR^+^ breast cancer samples (n = 119) were estimated using immunohistochemistry (IHC) to determine its association with the clinicopathological features, tamoxifen resistance, and survival outcomes. Western blotting (WB) and quantitative reverse transcriptase polymerase chain reaction (qRT-PCR) analysis was performed to estimate the MENA protein and mRNA levels in the tamoxifen-sensitive and -resistant HR^+^ breast cancer cell lines. Furthermore, CCK8, colony formation, and the transwell invasion and migration assays were used to analyze the effects of MENA knockdown on the biological behavior and tamoxifen sensitivity of the HR^+^ breast cancer cell lines. Xenograft tumor experiments were performed in the nude mice to determine the tumor growth rates and tamoxifen sensitivity of the control and MENA knockdown HR^+^ breast cancer cells in the presence and absence of tamoxifen treatment. Furthermore, we estimated the growth rates of organoids derived from the HR^+^ breast cancer patients (n = 10) with high and low MENA expression levels when treated with tamoxifen.

**Results:**

HR^+^ breast cancer patients with low MENA expression demonstrated tamoxifen resistance and poorer prognosis compared to those with high MENA expression. Univariate and multivariate Cox regression analysis demonstrated that MENA expression was an independent predictor of tamoxifen resistance in patients with HR^+^ breast cancer. MENA knockdown HR^+^ breast cancer cells showed significantly reduced tamoxifen sensitivity in the in vitro experiments and the in vivo xenograft tumor mouse model compared with the corresponding controls. Furthermore, MENA knockdown increased the in vitro invasion and migration of the HR^+^ breast cancer cells. HR^+^ breast cancer organoids with low MENA expression demonstrated reduced tamoxifen sensitivity than those with higher MENA expression. Mechanistically, P-AKT levels were significantly upregulated in the MENA-knockdown HR + breast cancer cells treated with or without 4-OHT compared with the corresponding controls.

**Conclusions:**

This study demonstrated that downregulation of MENA promoted tamoxifen resistance in the HR^+^ breast cancer tissues and cells by enhancing the AKT signaling pathway. Therefore, MENA is a promising prediction biomarker for determining tamoxifen sensitivity in patients with HR^+^ breast cancer.

**Supplementary Information:**

The online version contains supplementary material available at 10.1186/s13062-024-00464-3.

## Introduction

Globally, breast cancer (BC) is the most common malignancy and the main cause of cancer-related deaths among women [1]. BC is classified into four subtypes: luminal A, luminal B, HER 2 overexpression, and triple negative breast cancer. These subtypes are based on the expression levels of the estrogen receptor (ER), progesterone receptor (PR), human epidermal growth factor receptor 2 (Her-2), antigen Ki-67, and other protein molecules. The accurate subtype classification is necessary for precisely determining the prognosis and the optimal treatment strategy for patients with breast cancer [2]. Hormone receptor-positive (HR^+^) is the most common molecular type of breast cancer and includes ER-positive (ER^+^) and/or PR-positive (PR^+^) breast cancer [3, 4]. Currently, selective ER modulators (SERM), selective ER degraders (SERD), aromatase inhibitors (AIS), ovarian function inhibitors (OFS), mTOR inhibitors, and cell Cyclin-dependent kinase 4/6 (CDK4/CDK6) inhibitors are the main endocrine and targeted therapies for HR^+^ breast cancer patients [5–7]. Tamoxifen (TAM) was the first SERM drug approved by the US Food and Drug Administration (FDA) for clinical treatment. The structure of TAM is comparable to estrogen. Therefore, TAM significantly increases the recurrence times and reduces the mortality rates of HR^+^ breast cancer patients by competitively inhibiting the binding of estradiol to the estrogen receptor (ER) [8, 9]. However, approximately 40% of BC patients receiving tamoxifen treatment demonstrate drug resistance, which is associated with tumor recurrence, metastasis, and poor prognosis [6]. BC patients with tamoxifen resistance (TAMR) are treated with aromatase inhibitors (AIs), fluoxetine, CDK4/6 inhibitors, and other second-line medications. However, these interventions increase the disease-free survival (DFS) rates in BC patients with TAMR, but do not significantly improve their overall survival (OS). Tamoxifen resistance is associated with a multitude of factors, including *CYP2D6* gene polymorphisms, androgen receptor expression levels, HER-2 expression levels, G protein-coupled estrogen receptors, microRNAs (miRNAs), long non-coding RNAs (lncRNAs), and an array of molecular signaling pathways such as the estrogen receptor (ER) signaling pathway, PI3K-AKT-mTOR signaling pathway, receptor tyrosine kinase (RTK) signaling pathways involving HER2, EGFR, FGFR, and IGF1R, and the Hedgehog (HH) signaling pathway [10]. Currently, understanding the mechanisms of TAMR is a major focus area of breast cancer research. It is plausible that unraveling these mechanisms would lead to the identification of new and effective clinical biomarkers and therapeutic targets. Moreover, there is a lack of precise clinical indicators of TAM sensitivity and effective therapeutic strategies to overcome TAMR. Therefore, there is an urgent need to determine the mechanisms underlying TAMR and identify novel therapeutic targets to counteract TAM resistance and improve the survival rates of HR^+^ breast cancer patients.

Mammalian enabled (MENA) protein, also known as ENAH or hMENA is involved in the assembly and dynamics of the cytoplasmic actin networks [11]. MENA belongs to the enabled/vasodilator stimulated phosphoprotein (Ena/VASP) protein family and is encoded by the *MENA* gene on chromosome 1 [12]. Alternative splicing generates five different MENA transcripts, namely, MENA^+^, MENA^++^, MENA^+++^ (MENAINV), MENA^11a^, and MENA^Δv6^. Therefore, the expression patterns of the alternate MENA transcripts vary between different molecular types of breast cancer. MENA^11a^ is highly expressed in the HR^+^ breast cancer cell lines such as MCF7 and T47D, whereas MENA^ΔV6^ and MENA^INV^ are significantly expressed in the invasive breast cancer cell lines, including MDA-MB-231 and BT549 [13–15]. Currently, clinical studies investigating the associations of MENA and its splice variants with breast cancer estimate the expression levels of MENA (all MENA transcripts in total) in the cancer tissues [11]. Several studies have suggested that the expression of MENA/MENA^INV^ and MENA^11a^ promoted endocrine therapy resistance by enhancing or suppressing the activation of EGF-related signaling pathways [16, 17]. However, the relationship of MENA and its splice variants with TAMR in HR^+^ breast cancer patients have not been reported to date.

In this study, we investigated whether MENA was an effective biomarker for determining tamoxifen sensitivity in patients with HR^+^ breast cancer. Towards this, we estimated MENA expression levels in the tumor tissues of 119 HR^+^ breast cancer patients who underwent tamoxifen treatment. Survival analysis was performed to determine the association between MENA expression levels and the prognostic outcomes in these patients. Furthermore, we investigated the role of MENA in the proliferation, migration, invasion, and tamoxifen sensitivity of the BC cells by performing in vitro functional assays and in vivo experiments with nude mice using the control and MENA knockdown MCF7 and T47D cells. Finally, we investigated the signaling pathways downstream of MENA expression that modulate tamoxifen resistance or sensitivity in HR^+^ breast cancer.

## Results

### Low expression of MENA is associated with tamoxifen resistance and poor prognosis in the HR^+^ breast cancer patients

**Fig. 1:**
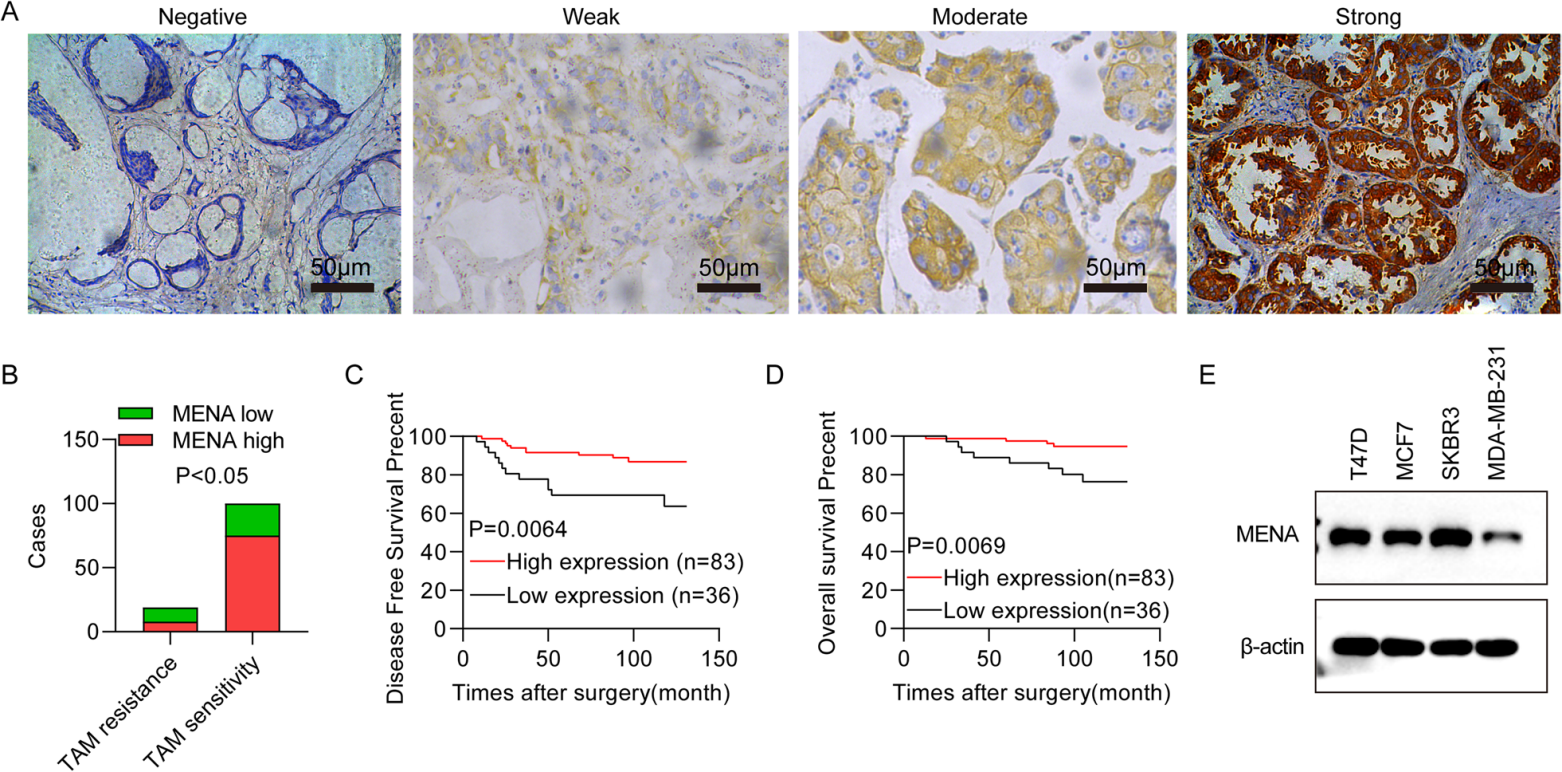
The association of MENA expression levels with tamoxifen resistance and prognosis of HR^+^ breast cancer patients (n = 119). **A** Representative immunohistochemical staining images show (a) negative ( −), (b) weak positive ( +), (c) medium positive (+ +) and (d) strong positive (+ + +) MENA expression in the BRCA tissues (Scale bar: 50 µm). **B** The statistic of A. **C**-**D** Kaplan–Meier survival curve analysis results show the association between MENA expression levels and **C** DFS, as well as **D** OS of the HR^+^ breast cancer patients (n = 119). **E** Expression of MENA in SKBR3, T47D, MDA-MB-231 and MCF7 cells

We first analyzed the correlation between MENA expression and tamoxifen resistance as well as the prognosis of HR^+^ breast cancer patients (n = 119). Based on MENA expression in the cancer tissues, the HR^+^ breast cancer patients were divided into low MENA (n = 36) and high MENA (n = 83) expression groups (Table 1). The expression of MENA protein was localized to the cytoplasm of the breast cancer cells in 76.47% (91/119) of the HR^+^ breast cancer patients (Fig. 1A, B). The median follow-up time for these patients was 110 months (range 9–129 months). During this follow-up period, the low MENA expression group showed higher rates of tamoxifen resistance (11/36 vs. 8/83; 30.56% vs. 9.64%), recurrence and metastasis (12/36 vs. 10/83; 33.33% vs. 12.05%), and mortality (8/36 vs. 4/83; 22.22% vs. 4.82%) compared with the high MENA expression group. HR^+^ breast cancer patients with low MENA expression levels were associated with shorter disease-free survival (DFS, P = 0.0064, Fig. 1C) and overall survival (OS, *P* = 0.0069, Fig. 1D) than those with high MENA expression levels. MENA expression levels were not associated with clinical characteristics such as patient age, tumor stage, tumor size, and lymph node metastasis (Table 1). Univariate and multivariate Cox regression analyses showed that low MENA expression was an independent predictor of tamoxifen resistance in the HR^+^ breast cancer patients (*P* = 0.003; Table 2). Finally, we detected the expression of MENA expression in BC cells. As showed in Fig. 1E, MENA was highly expression in SKBR3, T47D and MCF7 cells, while lower expression in MDA-MB-231 cells.

**Table 1:** The correlation between MENA expression levels and the clinical characteristics of the 119 HR^+^ breast cancer patients

Characteristic	Total (cases)	-MENA expression (cases)		χ2	*P* value
		High	Low		
All	119	83	36		
*Age (years)*					
< 50	94	66	28	0.046	0.830
≥ 50	25	17	8		
*Tumor size*					
T1 + T2	97	68	29	0.031	0.859
T3 + T4	22	15	7		
*Local lymph node metastasis*					
N0	50	32	18	1.350	0.245
N1-N3	69	51	18		
*TNM stage*					
I + II	82	59	23	0.607	0.436
III	37	24	13		
*ER expression*					
0–1 +	24	17	7	0.170	0.897
2–3 +	95	66	29		
*Her-2 expression*					
Over express	35	25	10	0.066	0.797
Low express	84	58	26		
*Tumor classification*					
Luminal A	62	43	19	0.009	0.922
Luminal B + Her-2 overexpression (HR +)	57	40	17

**Table 2:** Summary of the results from the univariate and multivariate COX proportional hazard model to determine factors influencing tamoxifen resistance in the HR^+^ breast cancer patients

Characteristic	Univariate		Multivariate	
	HR (95% CI)	P-value	HR (95% CI)	P-value
All				
*Age (years)*				
< 50	1.344 (0.484–3.730)	0.571		
≥ 50				
*Tumor size*				
T1 + T2	1.227 (0.407–3.696)	0.716		
T3 + T4				
*Local lymph node metastasis*				
N0	1.300 (0.512–3.302)	0.581		
N1-N3				
*TNM stage*				
I + II	2.176 (0.884–5.356)	0.091		
III				
*Her-2 expression*				
Over express	1.429 (0.563–3.630)	0.453		
Low express				
Tumor classification				
Luminal A	0.396 (0.151–1.042)	0.061		
Luminal B + Her-2 overexpression (HR +)				
*-MENA expression*				
Low	0.278 (0.112–0.693)	0.006	0.268 (0.104–0.691)	0.006
High				

### MENA expression is reduced in the tamoxifen-resistant HR^+^ breast cancer cells

**Fig. 2:**
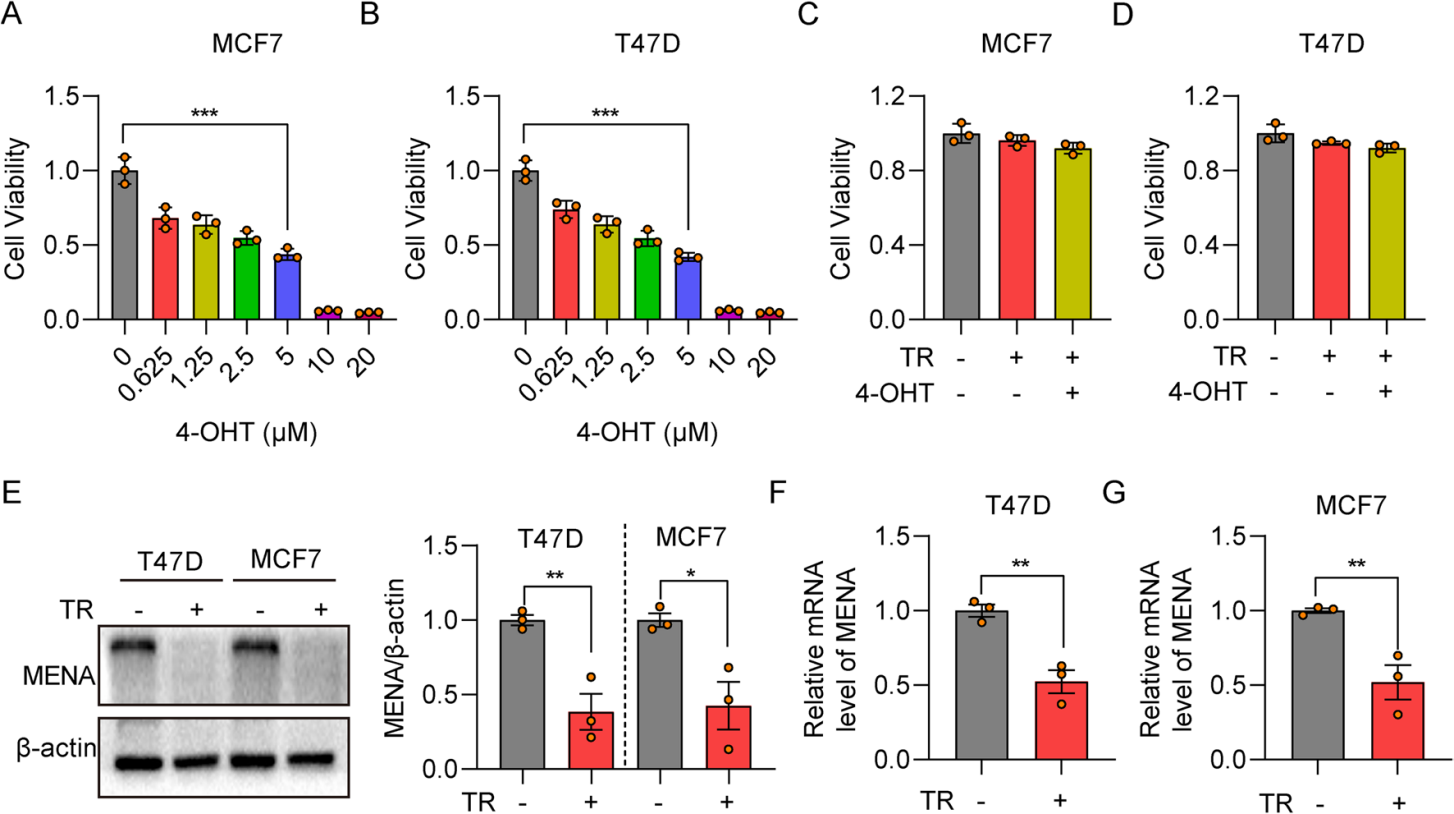
MENA is downregulated in the tamoxifen-resistant HR.^+^ BRCA cells. **A**-**B** CCK-8 assay results show the viability status of MCF7 and T47D cells treated with different concentrations of 4-OHT (0–20 µM) for 4 days. The viability of 4-OHT-treated cells was normalized to the control cells. **C**-**D** CCK-8 assay results show the viability status of MCF7, MCF7-TR, T47D, and T47D-TR cells cultured with 4-OHT for 4 days. Viability of the parental strains (MCF-7 and T47D) cultured in 4-OHT-free media was set as 1. **E** Western blot analysis of MENA protein levels in the MCF7, MCF7-TR, T47D, and T47D-TR cells. Β-actin was used as a loading control. The left panel shows the representative image of the western blot. The right panel shows the relative MENA protein expression levels calculated for the 4 cell lines from the blot. The relative expression of MENA to β-actin in the parental strains (MCF7 or T47D) was set to 1.0. **F**-**G** RT-qPCR analysis results show the relative MENA mRNA levels in the T47D, T47D-TR, MCF7 and MCF7-TR cells. The relative MENA mRNA levels in the parental strains (MCF-7 or T47D) were set to 1.0. *p < 0.05, **p < 0.01, ***p < 0.001

Next, to further verify the relationship between MENA expression and tamoxifen resistance, we generated tamoxifen-resistant HR^+^ breast cancer cell lines. CCK-8 assay results showed that the IC_50_ value for 4-OHT in the MCF7 and T47D HR^+^ breast cancer cell lines was 5 μM (Fig. 2A, B). Therefore, we used 5 μM 4-OHT to generate 4-OHT-resistant HR^+^ breast cancer cell lines, MCF7-TR and T47D-TR. CCK-8 assay results confirmed the tamoxifen resistance status of the, MCF7-TR and T47D-TR cells (Fig. 2C, D). Then, we compared the MENA mRNA and protein expression levels in the 4-OHT-sensitive HR^+^ breast cancer cells (MCF7 and T47D) and the 4-OHT-resistant HR^+^ breast cancer cells (MCF7-TR and T47D-TR). MENA protein levels were significantly decreased in the MCF7-TR and T47D-TR cells compared to the MCF7 and T47D cells, respectively (Fig. 2E). RT-qPCR analysis results also showed that the MENA mRNA levels were significantly reduced in the T47D-TR and MCF7-TR cells compared to the MCF7 and T47D cells, respectively (Fig. 2F, G). These results demonstrated that MENA expression was reduced in the tamoxifen-resistant HR^+^ breast cancer cells compared to the tamoxifen-sensitive HR^+^ breast cancer cells.

### MENA knockdown enhances invasion, migration, and tamoxifen resistance of the HR^+^ breast cancer cells

**Fig. 3:**
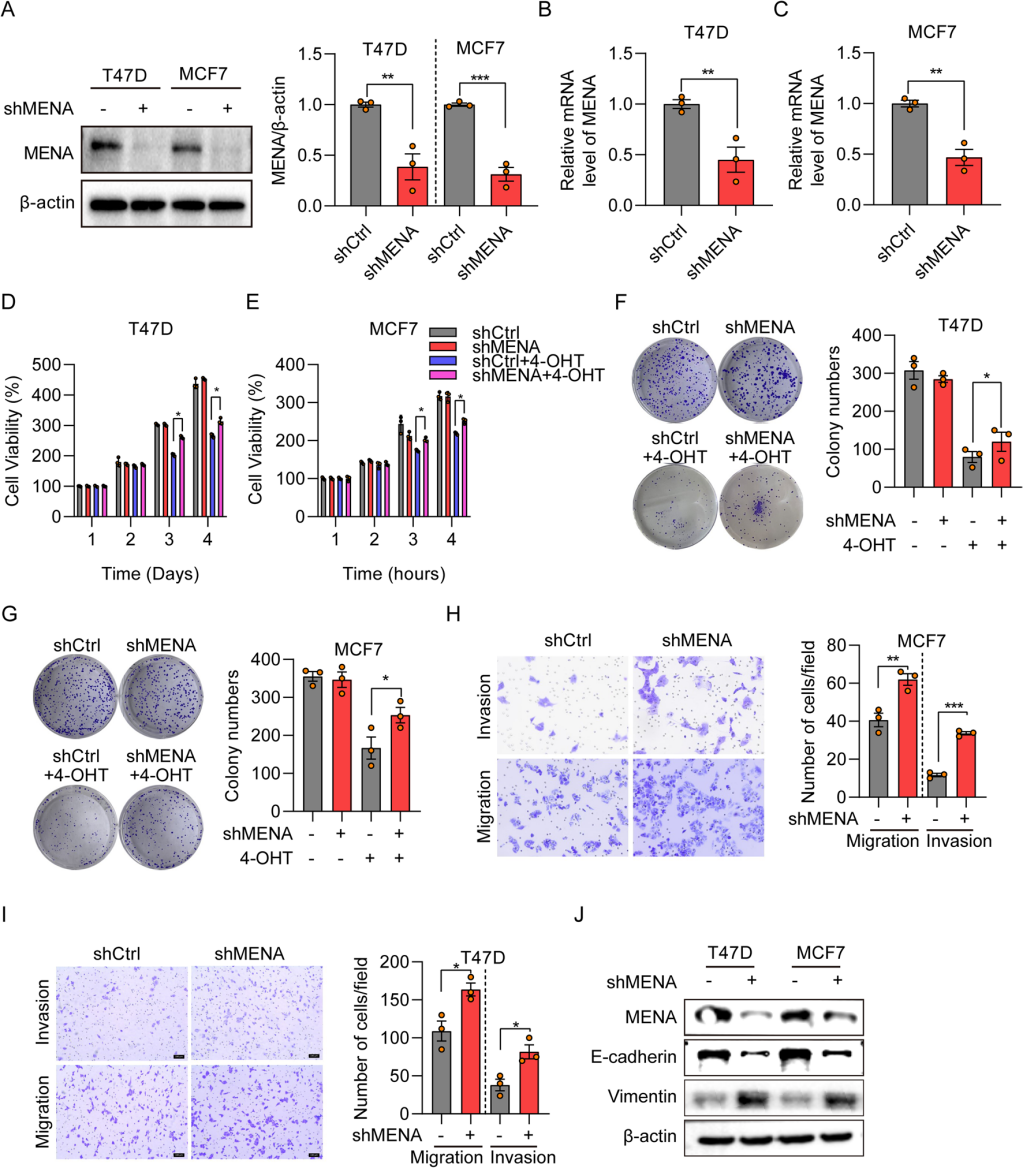
MENA knockdown increases invasion, migration, and tamoxifen resistance of the HR.^+^ BC cells. **A** Western blot analysis results show MENA protein expression levels in the shNC- and shMENA-transfected MCF7 and T47D cells. **B**-**C** RT-qPCR analysis results show the relative MENA mRNA levels in the shNC- and shMENA-transfected MCF7 and T47D cells. **D**-**E** CCK8 assay results show the viability status of the shNC- and shMENA-transfected MCF7 and T47D cells treated with or without 5 μm 4-OHT. **F**, **G** Representative images and quantitative analysis of the colony formation assay results show the total number of colonies formed by the shNC- and shMENA-transfected MCF7 and T47D cells treated with or without 5 μm 4-OHT. **H**-**I** Representative images and quantitative analysis of the transwell migration and invasion assays show the migration and invasion abilities of the shNC- and shMENA-transfected MCF7 and T47D cells treated with or without 5 μm 4-OHT. **J** Western blot analysis results show E-cadherin and Vimentin protein expression levels in the shNC- and shMENA-transfected MCF7 and T47D cells.*p < 0.05; **p < 0.01; ***p < 0.001; n = 3

Next, we investigated the effects of MENA knockdown on the biological behavior of HR^+^ breast cancer cells using MENA-specific shRNA. Western blot (Fig. 3A) and RT-qPCR (Fig. 3B-C) analyses confirmed the knockdown efficiency of MENA in the MCF7 and T47D cells. In our CCK-8 experiments, we detected no significant statistical disparities in the proliferation capacities of cells exhibiting diverse levels of MENA expression. Nevertheless, in the presence of tamoxifen, cells with distinct MENA expression levels exhibited a uniform deceleration in their proliferation capabilities. Notably, within the MENA knockdown group, cell proliferation surpassed that of the control group, signifying a statistically significant distinction. (Figs. 3D, E). The colony formation assay results also showed that MENA knockdown decreased sensitivity of the MCF-7 and T47D cells to tamoxifen (Figs. 3F, G). Furthermore, transwell assay results showed that MENA knockdown promoted migration and invasiveness of the MCF7 and T47D cells (Fig. 3H, I). Moreover, western blot assay indicated that the E-cadherin was down-regulated, while, vimentin was up-regulated after MENA silencing (Fig. 3J). These results suggested that reduced MENA expression correlated with increased proliferation, metastasis, and tamoxifen resistance of the HR^+^ breast cancer cells.

### Knockdown of MENA decreases in vivo tamoxifen sensitivity of the HR^+^ breast cancer cells

**Fig. 4:**
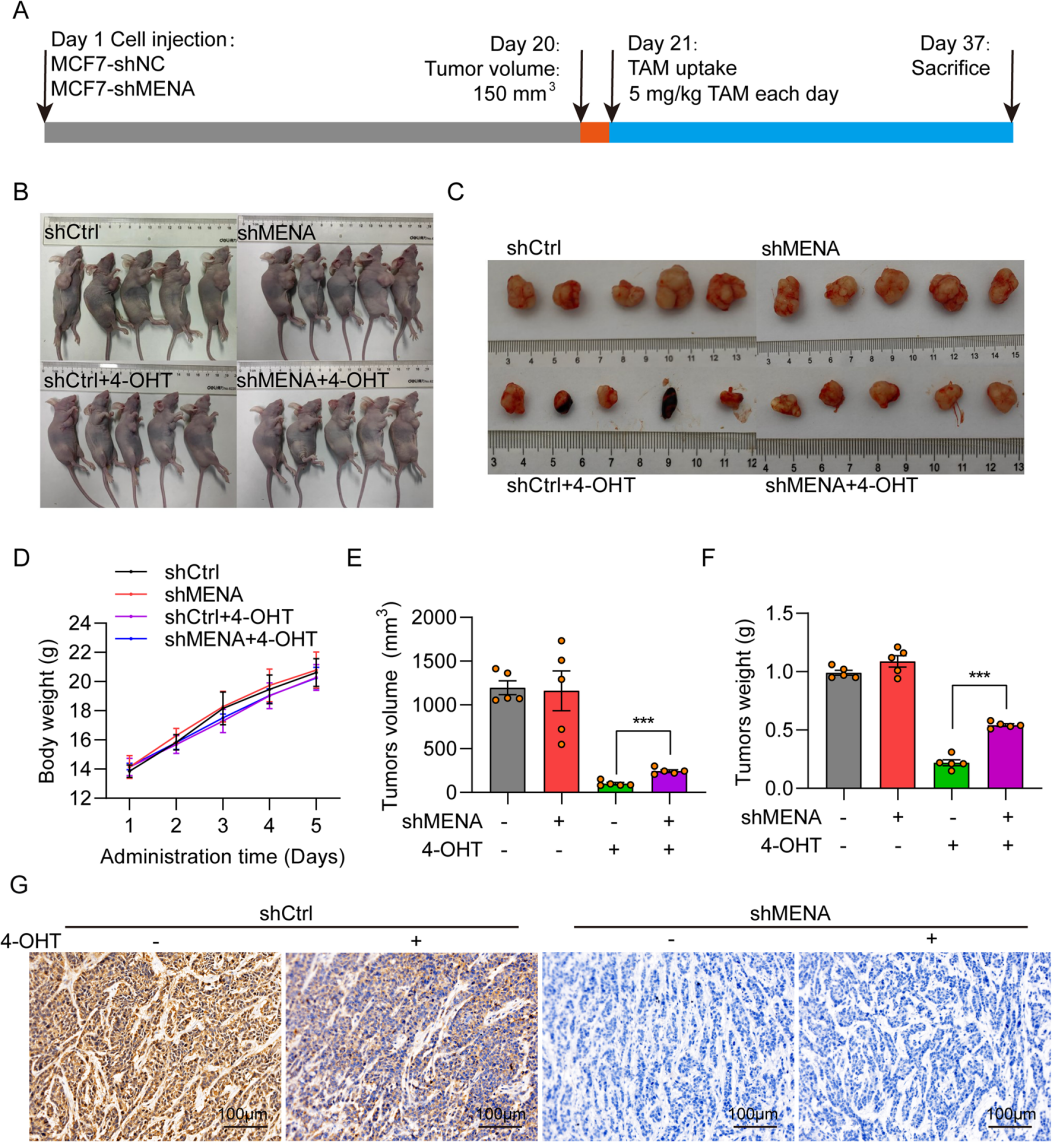
MENA knockdown confers in vivo resistance against tamoxifen in the HR.^+^ breast cancer cells. **A** The in vivo study design of the xenograft tumor growth experiment in nude mice. The tumor-bearing mice were sacrificed on day 43. **B** Representative photographs of the xenograft tumor-bearing mice belonging to the MCF7-shNC, MCF7-shNC + TAM, MCF7-shMENA, and MCF7-shMENA + TAM groups. **C** Representative images of the xenograft tumors isolated from the MCF7-shNC, MCF7-shNC + TAM, MCF7-shMENA, and MCF7-shMENA + TAM groups of mice at the final time point. **D** The growth curve of the nude mice belonging to the MCF7-shNC, MCF7-shNC + TAM, MCF7-shMENA, and MCF7-shMENA + TAM groups based on the tumor sizes between days 0–16 after injection of the tumor cells and treatment with or without tamoxifen. **E**, **F** Histograms show the **E** tumor volumes and **F** and tumor weights of the MCF7-shNC, MCF7-shNC + TAM, MCF7-shMENA, and MCF7-shMENA + TAM groups of mice at the end-point. **G** The NENA protein level in MCF7-shNC, MCF7-shNC + TAM, MCF7-shMENA, and MCF7-shMENA + TAM groups detected by IHC. Bar = 100 μm. **p < 0.01; ***p < 0.001; n = 5

We analyzed the in vivo effects of MENA knockdown in the HR^+^ BC cells on the xenograft tumor growth in the female nude mice when treated with or without tamoxifen. The xenograft tumor protocol for the animal experiments is outlined in Fig. 4A. The body size and weight of MCF7-shMENA and MCF7-shNC mice were shown in Fig. 4B, D. The sizes, volumes, and weights of the xenografted tumors were comparable between the MCF7-shMENA mice and the MCF7-shNC mice (Fig. 4C,E–F). However, when we compared the xenograft tumors in mice treated with tamoxifen, the tumor volume (243.68 + 53.67 mm^3^) (*p* < 0.001) and tumor weights (0.34 + 0.04 g) (*p* < 0.01) in the MCF7-shMENA + TAM group mice were significantly larger than those of the MCF7-shNC + TAM mice (Fig. 4E, F). IHC assay showed that the MENA was downregulated in both shMENA and shMENA + 4-OHT groups in xenograft tumor (Fig. 4G and Additional file 1: Fig. S1).

These results demonstrated that MENA knockdown did not alter the proliferation rates of the MCF7 tumor cells, but reduced their sensitivity to tamoxifen. The results of the in vivo xenograft tumor growth experiments in the nude mice were consistent with the results from the in vitro experiments.

### HR^+^ breast cancer organoids with low MENA expression levels show reduced sensitivity to tamoxifen

**Fig. 5:**
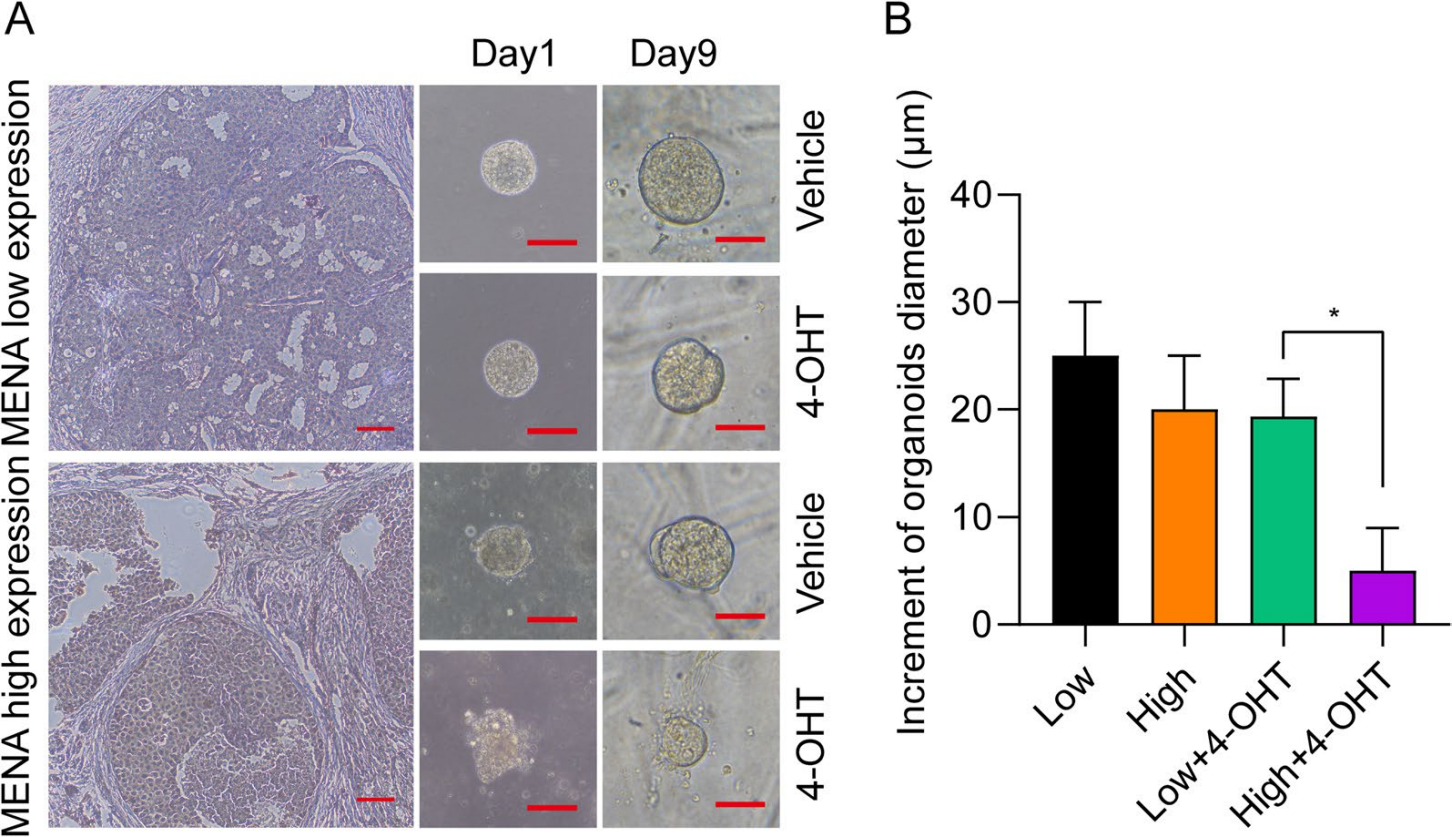
Growth of organoids derived from the HR^+^ breast cancer tissues with high or low MENA expression levels under tamoxifen treatment conditions. Organoids with a diameter of 50–100 μm were selected for the tamoxifen treatment experiments. The growth of organoids with different MENA expression levels was estimated after treatment with tamoxifen. **A** Representative images show the growth of organoids derived from HR.^+^ breast cancer tissues with high or low MENA expression levels on days 1, and 9 after treatment with or without 4-OHT. The maximum diameter of the corresponding organoids was measured. **B** Histogram shows the changes in the maximum organoid diameter after 9 days of culture in the low MENA, high MENA, low MENA + 4-OHT, and high MENA + 4-OHT groups. Bar = 100 μm. *P < 0.05

We then used 10 ER + breast cancer tissues obtained from patients that hadn’t undergone clinical radiotherapy and chemotherapy to perform organoid-like cultures and drug test experiments. This strategy was used to eliminate the impact of surgical methods and differential chemotherapy schemes on the follow-up endocrine treatment of patients with HR^+^ breast cancer. Immunohistochemistry (IHC) was used to analyze the expression levels of MENA in these 10 samples. IHC results showed that MENA expression levels were low in 3 cases and high in 7 cases (Fig. 5A). There was no significant difference in the growth rate of the organoid tissues derived from the ER + breast cancer tissues with high and low MENA expression levels (Fig. 5B). Tamoxifen treatment decreased the growth rate of all the organoids, but the sizes of the organoids belonging to the low MENA expression group were significantly larger than those in the high MENA expression group (Fig. 5B). These results demonstrated reduced tamoxifen sensitivity in the HR^+^ breast cancer cells with low MENA expression compared to those with high MENA expression.

### MENA enhances tamoxifen sensitivity of the HR + breast cancer by down-regulating the AKT signaling pathway

**Fig. 6:**
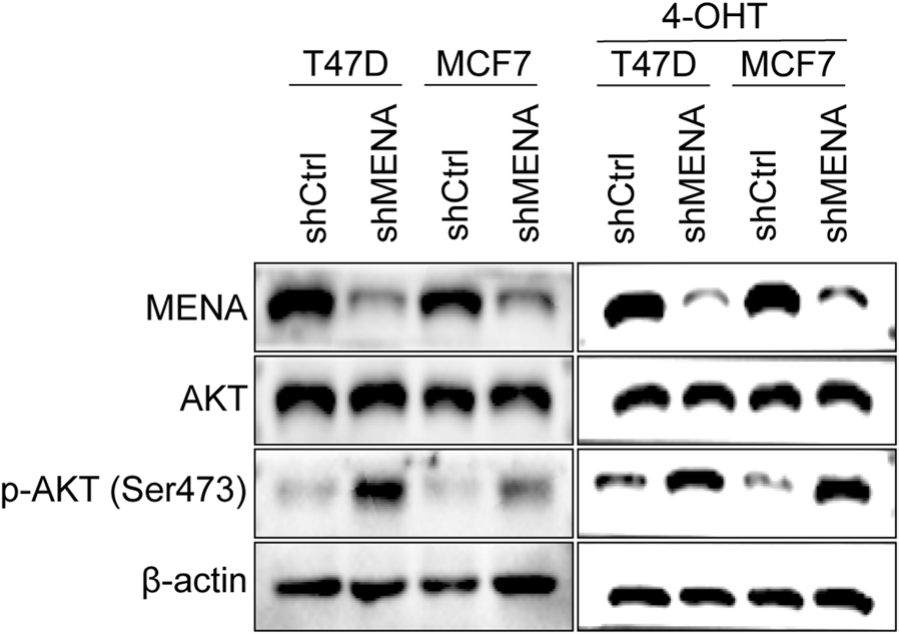
MENA knockdown promotes tamoxifen resistance in the HR^+^ breast cancer cells via AKT signaling pathway. Western blot analysis shows MENA, AKT, p-AKT (Ser473) and β-actin protein levels in the control and MENA-silenced T47D and MCF7 treated with or without 4-OHT cells

Finally, we investigated the mechanism by which MENA regulated tamoxifen resistance in the HR + breast cancer cells. We analyzed the status of the AKT signaling pathway in control and MENA-silenced T47D and MCF7, treated with or without 4-OHT cells. Western blot analysis showed that p-AKT (Ser473) protein levels were significantly higher in the MENA-silenced cells both in treated with or without 4-OHT compared with the control cells (Fig. 6). While the total AKT protein level was not change after MENA silencing. This result suggested that MENA knockdown increased tamoxifen resistance by enhancing the AKT signaling pathway.

## Discussion

Our study demonstrated that low MENA expression was associated with increased migration and invasion of the HR^+^ breast cancer cells. Furthermore, low MENA expression induced tamoxifen resistance (TAMR) in the HR^+^ breast cancer cells by enhancing the AKT pathway. The in vivo xenograft tumor model in the nude mice and the in vitro cultured organoids derived from the fresh HR^+^ breast cancer tissues confirmed that MENA expression was associated with the efficacy of tamoxifen treatment.

The overexpression of MENA in hepatocellular carcinoma is associated with tumor differentiation and clinical staging; moreover, MENA is an independent prognostic biomarker for determining the disease-free survival (DFS) times of patients with hepatocellular carcinoma [18]. MENA overexpression is associated with poor survival and aggressive phenotype in gastric cancer [19]. Our study showed that despite similar clinical and pathological features, HR^+^ breast cancer patients with low MENA expression levels were associated with tamoxifen resistance and poorer prognosis than those with higher MENA expression. Multivariate analysis demonstrated that MENA was an independent predictor of tamoxifen resistance in the HR^+^ breast cancer patients. These data suggested that MENA was a potential biomarker for predicting tamoxifen sensitivity or resistance in the HR^+^ breast cancer patients. Among all the different subtypes of breast cancer, HR^+^ breast cancer is associated with good prognosis [20],

There are multiple isoforms of MENA because of alternate splicing. These isoforms are differentially associated with tumor development and progression in a variety of cancers. For example, MENA^11a^ is overexpressed in the epithelioid breast cancer cells, whereas MENA and MENA^11a^ are upregulated in the HR^+^ breast cancer cells such as MCF7 and T47D [13]. We chose MCF7 and T47D breast cancer cells with high MENA^11a^ expression levels for this study. Since specific antibodies are not available for the MENA^11a^ subtype, we usedMENA antibodies in this study that can detect all the isoforms of MENA.

We then generated tamoxifen-resistant cell lines, MCF7-TR and T47D-TR, by treating the tamoxifen-sensitive parental cells, MCF-7 and T47D, with 4-OHT. Western blot analysis showed that MENA mRNA and protein levels were downregulated in the MCF7-TR and T47D-TR cells compared with the corresponding parental cells, MCF7 and T47D. The molecular weights of MENA (molecular weight: 88 kDa) and MENA^11a^ (molecular weight: 90 kDa) proteins are very close and are detected as a fused band in a western blot with the MENA antibody as shown in previous studies [21]. Balsamo et al. reported that co-expression of MENA^11a^ with MENA altered the functional characteristics of MENA because of the formation of hetero-tetramers with both isoforms [15].

We stably decreased MENA expression levels in the MCF7 and T47D cells by knocking down MENA using MENA-specific shRNAs. The knockdown of MENA did not alter the proliferation of shMENA-transfected MCF7 and T47D cells compared with the controls, but significantly decreased their sensitivity to tamoxifen. We validated these findings further by xenografting shNC- and shMENA-transfected MCF7 cells in the nude mice and analyzing the xenograft tumors in both groups. Weidmann et al. reported that breast cancer cells with high MENA^11a^ expression levels showed decreased invasiveness, but increased rates of proliferation because of high Ki67 expression levels [16]. CCK-8 assay results did not show any significant changes in the cell viability of shMENA-transfected MCF7 and T47D cells compared with the corresponding controls. However, the cell viability of shMENA-transfected MCF7 and T47D cells after tamoxifen treatment was slightly higher than the control group. This may be attributed to the physiological role of MENA in the cells. MENA is involved in the assembly and functioning of the cytoplasmic actin networks. Therefore, it is functionally associated with the motility and migration properties of cells but does not regulate cellular proliferation.

Recent advances in medical research have resulted in the development of organoid culture technology as an innovative experimental approach for drug testing and toxicity assessment because it mimics the 3-dimensional structural, functional, and genotypic properties of specific tissues [22, 23]. The organoid technique replicates the physiological and pathological processes in the human organs and provides a more authentic and dependable experimental framework for assessing drug sensitivity. Therefore, cultivation of organoid models with cancer cells or tissues derived from patients is increasingly being used for testing the efficacy of various drugs and to identify the optimal personalized treatment regimen for every patient [24, 25]. In this study, we used organoids derived from HR^+^ breast cancer patient tissues to verify the relationship between MENA expression levels and the efficacy of tamoxifen. Our data showed that the overall diameter of the organoid group with low MENA expression was significantly higher than that of the high MENA expression group in the presence of tamoxifen. This further confirmed that the tamoxifen sensitivity was significantly decreased in the HR^+^ breast cancer patients belonging to the low MENA expression group than those belonging to the high MENA expression group.

PI3K/AKT/mTOR pathway plays a significant role in sustaining endocrine resistance and is the target of many new drugs for patients with ER^+^ breast cancer [26, 27]. AKT, a serine/threonine kinase, is a downstream target of PI3K [28]. MENA promotes resistance against PI3K inhibitors in the HER2-overexpressing breast cancer cells via HER3 signaling pathways [29]. Our data showed that downregulation of MENA promoted tamoxifen resistance by enhancing PI3K-AKT signaling in the HR^+^ breast cancer cells.

Currently, molecular biomarkers that predict responses of breast cancer patients to tamoxifen therapy are not available [30]. MENA subtypes are potential biomarkers for predicting tumor progression in breast cancer and can be used to guide treatment strategy [31]. Our findings demonstrated for the first time that MENA regulated tamoxifen resistance in the HR^+^ breast cancer via AKT signaling. Therefore, MENA is a promising and novel clinical biomarker for predicting tamoxifen sensitivity in patients with HR^+^ breast cancer. Furthermore, MENA is also a target for reducing tamoxifen resistance and enhancing the efficacy of endocrine therapy in patients with HR^+^ breast cancer.

Our study has a few limitations. Firstly, our study included only 119 clinical cases from a single center. Therefore, further investigations are necessary in larger cohorts from multiple centers to confirm our findings. Secondly, we did not use MENA subtype-specific antibodies (e.g., MENA^11a^ specific antibodies). Therefore, it is not clear if any of the alternative MENA proteins are specifically involved in determining tamoxifen resistance. Thirdly, we did not determine whether P-AKT was regulated by 4-OHT, and How MENA regulates AKT signaling requires in-depth investigation.

## Conclusions

Our study demonstrated that low MENA expression was associated with increased migration, invasion, and tamoxifen resistance of the HR^+^ breast cancer cells by enhancing AKT signaling. Furthermore, our data showed that MENA was an independent predictor of tamoxifen sensitivity. Therefore, MENA is a potential target for reversing tamoxifen resistance in patients with HR^+^ breast cancer.

## Methods and materials

### Clinical samples

This study included 119 HR^+^ breast cancer patients that were treated at the Affiliated Cancer Hospital of Shantou University Medical College, Shantou, Guangdong Province, China, between June 2011 and May 2015. The inclusion criteria were as follows: (1) Female patients without menopause and aged between 20 and 55 years; (2) patients diagnosed with hormone receptor-positive (HR^+^) invasive breast cancer; (3) patients without a previous history of malignant tumors; (4) patients without distant metastasis; (5) patients that followed guidelines for the oral administration of tamoxifen as an adjuvant endocrine therapy after surgery. According to the guidelines by the American College of Clinical Oncology/College of American Pathologists (ASCO/CAP) for breast cancer, estrogen receptor (ER) and progesterone receptor (PR) positivity ≥ 1% is considered as positive [32, 33]. Her-2 testing was performed according to the 2007 guidelines of the American Society for Clinical Oncology and Pathology [34]. The HR^+^ endocrine therapy drug resistance standards for breast cancer were according to the ESO-ESMO Second Edition International Consensus Guidelines for Advanced breast cancer [35]. This study was approved by the Ethics Committee of the Affiliated Cancer Hospital of Shantou University Medical College (Approval No.2023105).

### Immunohistochemical staining

The breast cancer tissue slices were baked at 60 °C for 2 h, dewaxed in xylene for 10 min, and hydrated with different concentrations of ethanol for 5 min. Antigen retrieval was performed using the EDTA antigen repair solution. Then, endogenous peroxidase and non-specific antigens were blocked by incubating the tissue slices in 3% H_2_O_2_ solution and 10% goat serum for 30 min, respectively. Then, the slices were incubated overnight at 4 °C with the primary anti-MENA antibody (ab244423; Abcam; 1:500) followed by incubation with the secondary antibody at 37 °C for 30 min. The slices were then stained with the DAB solution (ZLI-9017, ZSGB-BIO, Beijing, China) and counter stained with hematoxylin for 3–4 min. Neutral resin was used to seal the slices on the slides and images were captured using the light microscope.

IHC stained sections were scored by two independent pathologists based on the previously reported protocols [36]. The sections were scored based on the percentage of cells with positive MENA staining and the intensity of MENA staining. Based on the percentage of positive MENA staining cells, the tissue sections were scored as follows: 0, no staining; 1, 0–25%; 2, 26–75%; 3, 76–100%. Based on the MENA staining intensity, the tissue sections were scored as follows: 0, no staining; 1, weak = light yellow; 2, medium = yellow brown; 3, strong = brown. Subsequently, the final MENA expression score was calculated for each patient by multiplying the score for the positive MENA staining and the score for the MENA staining intensity. The MENA expression score was between 0 and 9. Based on the final MENA expression score, the cases were classified into the following four categories: negative (-), 0–1; weakly expressed ( +), 2; moderately expressed (+ +), 3–5; and strongly expressed (+ + +), 6–9. The cases with negative or weak MENA expression were designated as “low MENA expression” and the cases with medium and strong MENA expression were designated as “high MENA expression.” If the pathological results for a case were inconsistent, it was not included in the group.

### Cell culture and establishment of tamoxifen-resistant HR^+^ breast cancer cells

MCF7 and T47D cells were cultured in DMEM medium (GIBCO) with 10% FBS (GIBCO) and 1% Penicillin–Streptomycin (Corning). Then, tamoxifen-resistant MCF7 and T47D HR^+^ breast cancer cell lines were generated by treatment with 4-hydroxytamoxifen (4-OHT), the active metabolite of tamoxifen, according to previously published protocols [37]. Briefly, MCF7 and T47D cells were cultured in DMEM medium with 5 μM 4-OHT for one year. The medium was changed every three days. The tamoxifen sensitivity of the cells was verified using the CCK-8 assay. The tamoxifen-resistant cells were then maintained by culturing in medium containing 0.1 μM 4-OHT.

### Western blotting

The total cellular proteins were extracted with the cell lysis buffer (P0013, Beyotime, Beijing, China) containing PMSF. The cell lysates were centrifuged at 12,000 rpm for 15 min at 4 °C. The total protein concentrations of the supernatants were estimated using the BCA kit (P0012S, Beyotime). Then, equal amounts of samples (30 μg) were separated on a 10% PAGE. The separated proteins were transferred onto the PVDF membranes (Merck Millipore). The membranes were blocked with 5% non-fat milk for 1 h. Subsequently, the membranes were incubated overnight at 4℃ with primary antibodies against MENA (ab244423, Abcam, 1:500), E-Cadherin (#3195, CST, 1:1000), Vimentin (sc6260, santa cruz, 1:1000), p-AKT (#4060, CST, 1:1000), AKT (ARG56418, Arigo, 1:1000), and β-actin (ab8227, Abcam, 1:1000). Then, they were incubated with the secondary antibodies at 37 °C for 120 min. The blots were developed using the ECL solution (P1050, Applygen, Beijing, China), and the protein bands were detected and quantified.

### Quantitative reverse transcriptase polymerase chain reaction (qRT-PCR)

Total RNA samples were extracted using the Trizol Reagent (Invitrogen). Reverse transcription of mRNA into cDNA was performed using the PrimeScript RT reagent kit (TaKaRa). Then, qPCR was performed using the SYBR® Premix Ex Taq™ II kit (TaKaRa) on the ABI 9700 PCR system (Applied Biosystems, Inc, USA). The PCR reaction conditions were as follows: 95℃ for 30 s, followed by 40 cycles of 95℃ for 5 s, 60 ℃ for 30 s, and 95 ℃ for 15 s. The primers used in this study were as follows: MENA (forward), 5 ‘- TCAAGGGTAAGGGAAACTGG-3’; MENA (reverse), 5′-TGGCTCACAAGTGGTCCTCC-3’; β-Actin (forward), 5′- AGCGAGCATCCCCCAAAGTT-3′; β-Actin (reverse), 5′- GGGCACGAAGGCTCATCATT-3′.

### CCK-8 assay

CCK-8 assay was used to estimate the viability of the breast cancer cells. Briefly, we seeded 2000 cells per 200 µl of culture medium per well in a 96-well plate for 0, 24, 48, 72, and 96 h. Then, we added 10 µl of CCK-8 solution (C0009, Beyotime) to each well at the defined time points and further incubated the cells at 37℃ for 2 h. The absorbance (OD value) was measured at a wavelength of 450 nm using an automatic microplate spectrophotometer (ELx800TM, BioTek Instruments, Inc, USA).

### Colony formation assay

For the cell colony formation assay, we seeded 500 cells in 2 ml of culture medium per well in a 6-well plate. After 2 weeks of colony formation, the culture medium was removed. The colonies were washed thrice with PBS. Then, the colonies were fixed with methanol for 20 min, stained with 0.1% crystal violet (Solarbio) for 30 min, and the number of colonies counted using a light microscope.

### Transwell migration and invasion assay

Transwell assays were used to estimate the in vitro invasion and migration status of the control and MENA-knockdown HR^+^ breast cancer cells. Briefly, the HR^+^ breast cancer cells in the logarithmic growth phase were harvested and starved in serum-free medium for 24 h. Subsequently, we seeded 5 × 10^4^ cells in 200 μl of serum-free DMEM in the upper chamber of the transwell. In the case of invasion assays, the upper transwell chamber was coated with 100 µl of diluted Matrigel (1:8 in DMEM medium). In the lower chamber, we added 600 μl of DMEM containing 20% FBS. The transwell chambers were then incubated for 24 h in the incubator. Subsequently, the cells in the upper chamber were wiped off with a cotton swab. Then, the migratory or invasive cells in the bottom chamber were fixed with methanol, stained with 0.1% crystal violet, photographed under the light microscope, and counted.

### Lentivirus-mediated MENA knockdown in the HR^+^ breast cancer cell lines

HR^+^ breast cancer cell lines, MCF7 and T47D, with high MENA^11a^ expression levels were selected for the knockdown experiments. Briefly, 5 × 10^4^ cells/ml were seeded into a 6-well plate and incubated at 37℃ for 24 h. Lentiviruses with shNC or shMENA were diluted to the desired concentration using the complete culture medium. Then, the culture medium was removed from the cells. Subsequently, the cells were incubated for 16 h with 1 ml of complete culture medium containing the lentivirus and the infection enhancing solution according to the manufacturer’s instructions. Subsequently, the cells were incubated for 48 h with medium containing 1 μg/ml puromycin to select the successfully transfected cells. The knockdown efficiency of MENA was verified by western blotting and RT-qPCR. The following shRNA sequences were synthesized for the knockdown of MENA knockdown by the Shanghai Jima Company (Shanghai, China): MENA shRNA (forward), 5 '- GCAAAAAGGGAGGAGCGACAA-3'; MENA shRNA (reverse), 5 '- GCAAGAGGAGAGCAAGCAA-3'.

### Animal experiments

We purchased twenty 3-week-old BALB/c nude mice from the Beijing Vital River Laboratory Animal Technology Co., Ltd. They were fed for one week with adequate food and water containing estrogen. Subsequently, MCF7-shMENA cells or MCF7-shNC cells (2.0 × 10^6^ cells in 100 µl medium per mouse) were injected into the right armpit of the nude mice (n = 10 per group). When the average volume of the tumor reached 500 mm^3^, five mice in each group were orally administered with 5 mg/kg tamoxifen each day. The mice were divided into the following four groups: MCF7-shNC; MCF7-shNC + TAM; MCF7-shMENA; and MCF7-shMENA + TAM. The length (L) and width (W) of the tumors, and the weights of mice were measured and recorded every 3 days. The tumor volume was calculated using the following formula: V = (L x W^2^)/2. At the end of the experiment, the mice were euthanized. The tumors were harvested and weighed. The animal experiments were approved by the Ethics Committee of the Cancer Hospital of Shantou University Medical College (Approval No. SUMC2022-587).

### Organoid experiments

We obtained 10 new tumor tissue samples of HR^+^ breast cancer patients that were treated at the Affiliated Cancer Hospital of the Shantou University Medical College between October 2022 and November 2022 for organoid culturing and drug efficacy experiments. The inclusion criteria were the same as described for the 119 patients above. The Ethics Committee of the Affiliated Cancer Hospital of the Shantou University Medical College approved this study (Approval No.2023057).

The tumor tissue samples were placed in a 10 cm glass petri dish filled with D-BSA and cut into pieces (0.5–1 mm^3^) with a scalpel. The tissue fragments were transferred into a 15 ml centrifuge tube. The supernatant was discarded, and the tissue fragments were resuspended in 10 ml D-BSA. The above steps were repeated four times. Finally, the tissue fragments were resuspended in 5 ml of organoid culture medium followed by the addition of 250 μL collagenase (1 mg/ml) and 5μL ROCK inhibitor (10 μM). The tissue fragments were digested at 37 °C in a table concentrator for 1 h. Then, 1 ml FBS was added into the test tube to terminate the collagenase digestion. The digested interstitial fluid was transferred into a 50 ml centrifuge tube and centrifuged at 450 × g for 10 min at 8 °C. Then, the cell sediment was resuspended in 100 μl BME (PM151216, Pricella, Wuhan, China). Subsequently, 2–3 drops of BME were mixed with the organoid in a 24-well plate. The plate was inverted and placed in an incubator at 37 °C for 30 min. Then, 500 μl of the organoid culture medium was added into the 24-well plate and transferred into a humidified incubator maintained at 37 °C and 5% CO_2_. Fresh culture medium was added every 2–4 days, and the organoids were passaged every 7–21 days according to their growth status.

We used 50 μm organoids to perform the drug sensitivity test. We added the organoid culture medium containing tamoxifen into the experimental hole. For the control group, the culture medium was changed every three days. In each hole, we selected an organoid of similar size as the observation object and captured photographs using an inverted open field microscope (4x, 10 × or 20 × objective lens) every 4 days to record the shape and size of the organoid. Finally, the changes in the maximum diameter of the organoids after different treatments were calculated.

### Statistical analysis

Statistical analyses were performed using the SPSS software (version 23.0; IBM Corp., USA) and the GraphPad Prism software (version 9.0; GraphPad Prism software, California, USA). Kaplan Meier survival curves and logarithmic rank test were used to analyze the relationship between MENA expression levels and overall survival (OS), and disease-free survival (DFS) of the HR^+^ breast cancer patients. Chi-squared test was used to analyze the relationship between the MENA expression levels and the clinicopathological characteristics of the patients. Univariate and multivariate COX regression analyses were used to determine whether MENA expression was an independent predictor of tamoxifen sensitivity and tumor progression in the HR^+^ breast cancer patients based on the hazard ratio and the 95% confidence interval. The statistical differences between two groups were compared using the T-test. The statistical differences between three or more groups were estimated using one way ANOVA. *P* < 0.05 was considered statistically significant.

### Supplementary Information

**Additional file 1. Fig. S1.** Protein level of MENA in all tumor samples by IHC.

## Data Availability

The original contributions presented in the study are included in the article. Further inquiries can be directed to the corresponding author.

## Abbreviations

MENA: Mammalian Ena homolog
BC: Breast cancer
ER: Estrogen receptor
PR: Progestogen receptor
Her-2: Epidermal growth factor receptor 2
HR +: Hormone receptor positive
SERM: Selective ER modulator
SERD: Selective ER modulator
AIS: Aromatase inhibitor
OFS: Ovarian function inhibitor
CDK4/CDK6: Cell Cyclin-dependent kinase 4/6
TAM: Tamoxifen
FDA: Food and Drug Administration
TAMR: Tamoxifen resistance
Ais: Aromatase inhibitors
DFS: Disease-free survival
OS: Overall survival
miRNAs: MicroRNAs
LncRNAs: Long non-coding RNAs
RTK: Receptor tyrosine kinase
HH: Hedgehog
