# Supplementary figures and images for: Mammalian enabled protein enhances tamoxifen sensitivity of the hormone receptor-positive breast cancer patients by suppressing the AKT signaling pathway
Source: Biol Direct. 2024 Mar 8;19:21. doi: 10.1186/s13062-024-00464-3 (PMC10921784; doi:10.1186/s13062-024-00464-3)

## Slide 1
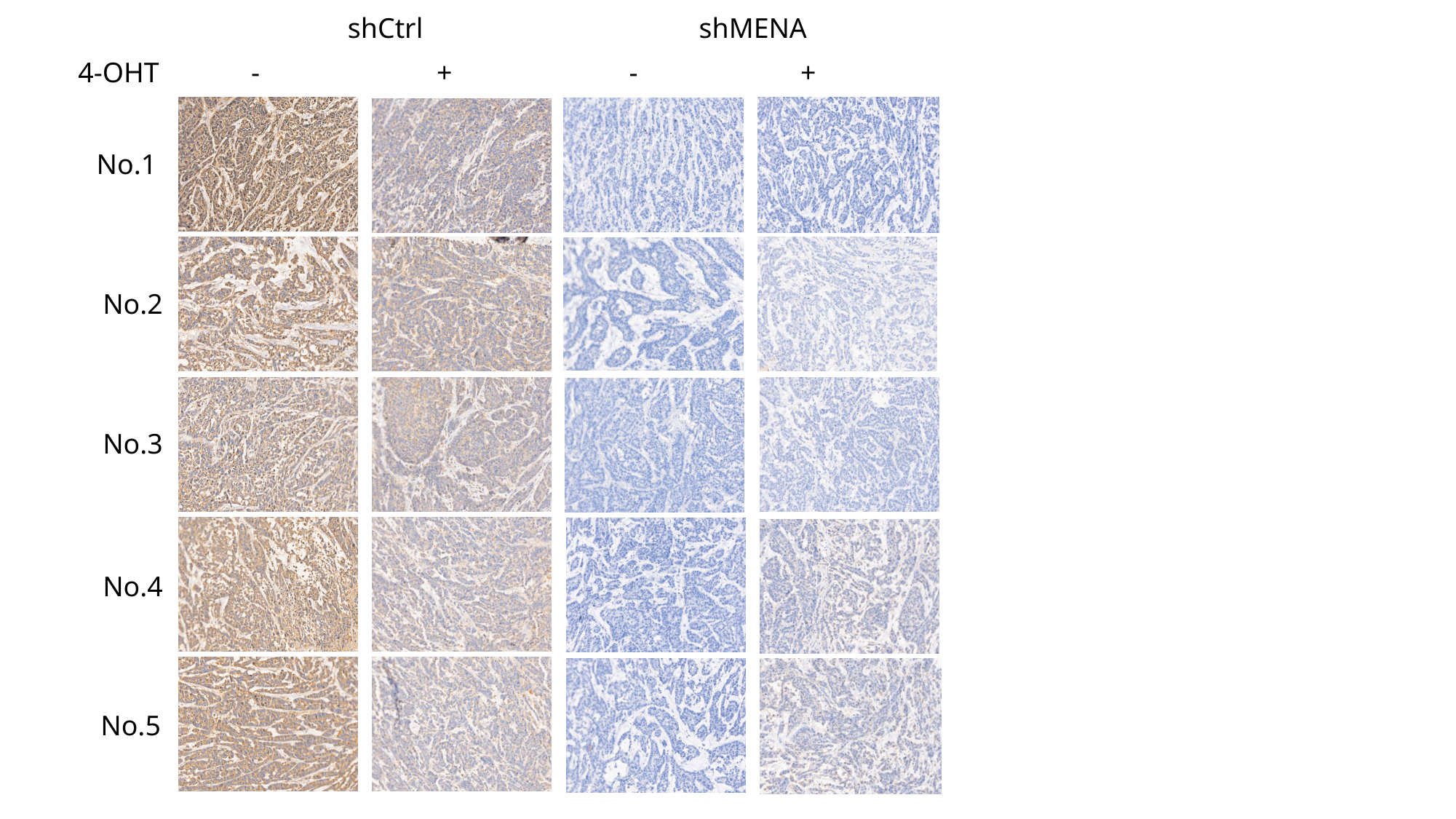

shCtrl shMENA
4-OHT - + - +
No.1
No.2
No.3
No.4
No.5

Supplement: Supplementary file 1 — Additional file 1. Fig. S1. Protein level of MENA in all tumor samples by IHC. [file 13062_2024_464_MOESM1_ESM.pptx]
